# Adaptive Iterative Dose Reduction Using Three Dimensional Processing (AIDR3D) Improves Chest CT Image Quality and Reduces Radiation Exposure

**DOI:** 10.1371/journal.pone.0105735

**Published:** 2014-08-25

**Authors:** Tsuneo Yamashiro, Tetsuhiro Miyara, Osamu Honda, Hisashi Kamiya, Kiyoshi Murata, Yoshiharu Ohno, Noriyuki Tomiyama, Hiroshi Moriya, Mitsuhiro Koyama, Satoshi Noma, Ayano Kamiya, Yuko Tanaka, Sadayuki Murayama, for the investigators of ACTIve Study Group

**Affiliations:** 1 Department of Radiology, Graduate School of Medical Science, University of the Ryukyus, Nishihara, Okinawa, Japan; 2 Department of Radiology, Osaka University Graduate School of Medicine, Suita, Osaka, Japan; 3 Department of Radiology, Shiga University of Medical Science, Otsu, Shiga, Japan; 4 Department of Radiology, Kobe University Graduate School of Medicine, Kobe, Hyogo, Japan; 5 Department of Radiology, Ohara General Hospital, Fukushima-shi, Fukushima, Japan; 6 Department of Radiology, Osaka Medical College, Takatsuki, Osaka, Japan; 7 Department of Radiology, Tenri Hospital, Tenri, Nara, Japan; 8 Center for Clinical Training, Fujieda Municipal General Hospital, Fujieda, Shizuoka, Japan; Mayo Clinic College of Medicine, United States of America

## Abstract

**Objective:**

To assess the advantages of Adaptive Iterative Dose Reduction using Three Dimensional Processing (AIDR3D) for image quality improvement and dose reduction for chest computed tomography (CT).

**Methods:**

Institutional Review Boards approved this study and informed consent was obtained. Eighty-eight subjects underwent chest CT at five institutions using identical scanners and protocols. During a single visit, each subject was scanned using different tube currents: 240, 120, and 60 mA. Scan data were converted to images using AIDR3D and a conventional reconstruction mode (without AIDR3D). Using a 5-point scale from 1 (non-diagnostic) to 5 (excellent), three blinded observers independently evaluated image quality for three lung zones, four patterns of lung disease (nodule/mass, emphysema, bronchiolitis, and diffuse lung disease), and three mediastinal measurements (small structure visibility, streak artifacts, and shoulder artifacts). Differences in these scores were assessed by Scheffe's test.

**Results:**

At each tube current, scans using AIDR3D had higher scores than those without AIDR3D, which were significant for lung zones (*p*<0.0001) and all mediastinal measurements (*p*<0.01). For lung diseases, significant improvements with AIDR3D were frequently observed at 120 and 60 mA. Scans with AIDR3D at 120 mA had significantly higher scores than those without AIDR3D at 240 mA for lung zones and mediastinal streak artifacts (*p*<0.0001), and slightly higher or equal scores for all other measurements. Scans with AIDR3D at 60 mA were also judged superior or equivalent to those without AIDR3D at 120 mA.

**Conclusion:**

For chest CT, AIDR3D provides better image quality and can reduce radiation exposure by 50%.

## Introduction

Iterative reconstruction techniques have gradually been applied to several multidetector-row computed tomography (MDCT) scanners, which recently became available due to increased computational power and created a new generation of reconstruction methods after conventional filtered back projection (FBP) and basic image filtering [1], [2]. Although the definitions of iterative reconstruction differ among CT manufacturers, iterative reconstruction typically involves multiple iteration cycles during the reconstruction process until final output images are created, and often enhances input images by using various algebraic models rather than simple noise reduction prior to the iterative cycles.

Each CT manufacturer has developed unique iterative reconstruction algorithms that have been used for chest CT imaging. These include Iterative Reconstruction in Image Space (IRIS) and Sinogram Affirmed Iterative Reconstruction (SAFIRE) by Siemens Healthcare [2]–[8], Adaptive Statistical Iterative Reconstruction (ASIR) and Model Based Iterative Reconstruction (MBIR) by GE Healthcare [9]–[18], and iDose algorithms by Philips Healthcare [19]–[21]. Adaptive Iterative Dose Reduction using Three Dimensional Processing (AIDR3D) is an iterative reconstruction method that was developed by Toshiba Medical Systems. Briefly, AIDR3D incorporates unique noise reduction processing, which includes statistical and scanner models for projection data, and multiple cycles of information syntheses with edge-handling, smoothing, and blending of original input images until final output images are created. AIDR3D is currently routinely provided with all major MDCT scanners from this manufacturer. Although the positive effects of AIDR3D have been investigated for some organs [22]–[24], only a few studies have investigated the advantages of AIDR3D for chest CT [25]–[27].

To the best of our knowledge, no previous study evaluated the effects of AIDR3D on clinical chest CT images that demonstrate several representative patterns of lung diseases using 320-row scanners. Further, only a few previous studies evaluated the effects of iterative reconstruction methods on chest CT images using multiple tube current settings [10], [14], [25]. Thus, we still believe that it would be meaningful to investigate the advantages of AIDR3D for chest CT images, particularly for comparing scan series at various tube current settings with and without AIDR3D.

Because AIDR3D can distinguish x-ray signals from image artifacts or noise more accurately than conventional reconstruction modes, it would be expected that noise and artifacts would be reduced and that image quality would be improved. This might also provide for substantial dose reductions in clinical CT scanning. Therefore, we hypothesized that by using multiple tube current settings, the improvements in image quality using AIDR3D would be clarified and that dose reductions might be possible.

Thus, the aims of this study were: (1) to investigate the positive effects of AIDR3D on chest CT images; and (2) to assess whether dose reductions were feasible for chest CT imaging using AIDR3D.

## Materials and Methods

This study was conducted as part of the Area-detector Computed Tomography for the Investigation of Thoracic Diseases (ACTIve) Study, an ongoing multicenter research project in Japan. The research committee of the study project outlined and approved our study protocols. The Institutional Review Board of each of the participating institutions approved this study: Kobe University, Osaka University, Tenri Hospital, Shiga University of Medical Science, and University of the Ryukyus.

Written informed consent was obtained from all enrolled subjects.

### Subjects

From January to March of 2012, a total of 103 subjects were initially enrolled for this study at the five participating institutions. Inclusion criteria were: (1) adult patients (≥ 20 years-old); (2) provided full informed consent; (3) planned plain chest CT as a part of routine clinical care for assessing known or suspected chest diseases, such as primary lung cancer, lung metastasis, emphysema, interstitial lung disease, or lymphadenopathy.

Exclusion criteria were: (1) pregnancy (n = 0); (2) lack of breath-holding (n = 2); (3) image artifacts caused by previous surgical procedures and a cardiac device (n = 1); (4) previous lobectomy or pneumectomy (n = 3); (5) lobar atelectasis (n = 2); and (6) data damaged during data transportation (n = 7).

Finally, 88 subjects were evaluated in this study. Forty-nine females and 39 males were included. Their mean age was 65 ± 13 years and their mean body weight was 59.2 ± 12.4 kg.

### Chest CT

During a single visit for each, the 88 subjects underwent plain chest CT (64-row helical mode) using identical 320-row MDCT scanners (Aquilion ONE, Toshiba Medical Systems, Otawara, Tochigi, Japan). Each subject was scanned three times using different tube currents (240, 120, and 60 mA) with the same rotation time (0.35 sec). Thus, the final current settings were 84, 42, and 21 mAs. Scanning field of view (FOV) was selected from two settings based on patient body habitus: 320 (medium) or 400 mm (large). Other scanning and reconstruction settings were fixed: tube voltage = 120 kVp; collimation = 0.5 mm; beam pitch = 0.828 (helical pitch, 53); imaging FOV = 320 mm; slice thickness = 1 mm (without image interval or overlapping); and reconstruction kernels: FC52 for lung and FC13 for mediastinum.

Scan data were converted to CT images using AIDR3D (‘standard’ setting) and a conventional FBP mode (Boost3D  =  without AIDR3D). Thus, 12 CT series with or without AIDR3D (6 series with the lung kernel and 6 series with the mediastinal kernel) were made for each patient. The AIDR3D mode did not require additional processing time; thus, CT images acquired with AIDR3D and Boost3D were created immediately after scanning was completed. All 1056 CT series (12 series×88 subjects) were anonymized and stored in a workstation viewer (ZIOstation2, Ziosoft Inc., Tokyo, Japan).

Radiation exposure was calculated using dose-length product (DLP) values, which were based on CT dose index volumes (CTDI vol). The effective dose was retrospectively calculated by multiplying the DLP values by a factor of 0.0145 [28].

### Qualitative image analysis - Lung

All qualitative analyses were made independently by three board-certified radiologists of the Japan Radiological Society (T.M., H.K, and O.H., 10, 11, and 20 years of experience in thoracic radiology). These observers were blinded to the scanning protocols and patients' information. The median scores of these three observers were used as the final scores.

CT series were displayed on diagnostic-grade LCD monitors with axial and reconstructed coronal views using a fixed lung window setting (level: −600 HU; width: 1600 HU). In total, 528 series (6 series×88 subjects) were randomly presented to the observers by assistants: one of six CT series for a single patient was randomly selected by assistants and given to an observer. This was followed by a series of six CT series for the next subject, which was selected randomly by the assistants. After all subjects were reviewed once during the first sub-session, the second sub-session started and one of the remaining five series was randomly selected and presented. Thus, the whole reading session consisted of six sub-sessions. Scan series for the same subject using different tube currents or different reconstruction modes did not appear simultaneously or contiguously during the reading session.

Using a 5-point scale from 1 (non-diagnostic) to 5 (excellent), the three observers independently evaluated overall image quality for three lung zones (upper, middle, and lower zones) based on image noise observed in the lung parenchyma. These three zones were determined by the aortic arch and the lower pulmonary vein. Also, when a subject had any one of four patterns of lung disease, including pulmonary nodule/mass (≥1 cm in diameter), emphysema, bronchiolitis, or diffuse lung disease, overall image quality for these disease patterns was scored using the 5-point scale. These disease patterns were detected and recorded prior to the reading session by another board certified radiologist of the Japan Radiological Society who had 11 years of experience with thoracic imaging and did not participate in the qualitative analysis.

Among the 88 subjects, 20 had 25 nodules or masses (solid lesion, n = 20; ground-glass opacity, n = 5), 18 had pulmonary emphysema, 12 had bronchiolitis (multiple centrilobular nodules), and 18 had a diffuse lung disease, such as interstitial pneumonia. During a reading session, information on the location and extent of the targeted disease was provided to the observers by assistants. The overall image quality of these diseases was determined based on the following points: (1) pulmonary nodule/mass: sharpness of tumor margins, visibility of internal structures, and artificial density heterogeneity caused by image noise or artifact; (2) emphysema: visibility of emphysematous change, and image noise in the emphysema space; (3) bronchiolitis: detectability and visibility of centrilobular nodules; and (4) diffuse lung disease: clearness of fibrotic or honeycombing changes, and image noise included in ground-glass opacity (GGO) or reticular shadow.

### Qualitative image analysis - Mediastinum

Axial images were displayed with a fixed mediastinal window setting (level: 40 HU; width: 400 HU). Using the same 5-point scale, the observers evaluated three image quality patterns: visibility of small structures; severity of streak artifacts; and influence of shoulder artifacts. Visibility of small structures was based on whether or not small lymph nodes or mediastinal vessels were clearly visualized. Streak artifacts were estimated based on the severity of radial streaks, typically observed in the heart spreading from the spine. Shoulder artifacts were defined as horizontal density inconsistency caused by bony structures around the shoulder joints, which sometimes creates artificial black zones in periclaviculer areas.

### Quantitative image analysis - Lung

All quantitative analyses were made using the same workstation as used for qualitative analyses by a single board-certified radiologist of the Japan Radiological Society (T.Y., 11 years of experience in thoracic radiology). Because the quantitative analyses required measuring comparable regions in the lungs and mediastinum based on their anatomical information, all 6 scan series with and without AIDR3D were displayed simultaneously and set side-by-side on the screen. Thus, this observer was aware of the scanning protocol for each scan series.

Images at the level of the lung apices, carina, inferior pulmonary veins, and lung bases were used for quantitative analysis of image quality. First, the scanner table locations corresponding to the four levels were determined on each scan series. Second, circular regions of interest (ROI) of 10 mm in radius were set on the right lung parenchyma. ROIs were carefully placed by the observer to avoid including pulmonary vessels or bronchi. All ROIs were then reproduced on the other CT series, and image noise at each ROI was determined as the SD of the CT values within the ROIs. Thus, a total of 2112 ROI measurements (6 series×4 levels×88 subjects) were made to determine image noise for scans with the lung kernel. Parenchyma of the left lung was not measured in this study, because it was expected that cardiac motion artifacts would artificially skew the measurements.

### Quantitative image analysis - Mediastinum

Similar to the lung measurements, circular ROIs of 10 mm in radius were placed in the aortic arch and the descending aorta by the same radiologist to measure the SD of the CT values. A total of 1056 ROI measurements (6 scans×2 levels×88 subjects) were made to investigate image noise for scans made with the mediastinal kernel.

### Phantom Study

A chest phantom (N1 Lungman, Kyoto-kagaku, Kyoto, Japan) was used to investigate the signal-to-noise ratio (SNR) and image sharpness on chest CT. The chest phantom had imitation soft tissues, including those of chest walls, heart, and aorta, which were constructed from uniform polyurethane resins. Imitation ribs, pulmonary vessels, and thoracic spine (vertebrae and discs) were also incorporated in the phantom (Figure S1). Imitation lungs were not available, thus presumed pleural cavities consisted of air. Two imitation spherical nodules (a solid nodule with 100 HU and a GGO nodule with -630 HU, 10 mm in diameter) were inserted in the pleural cavities. The phantom was scanned three times to create six scan series using the same scanning and reconstruction settings (240, 120, and 60 mA; with and without AIDR3D).

Based on a previous study that investigated the SNR of pulmonary nodules on clinical CT images [29], the SNR was obtained with the following methods using the same workstation that was used for the human study. On axial images with the mediastinal view setting (FC13), circular ROIs (8 mm in diameter) were placed in the imitation nodules, which were used to measure mean nodule attenuation and image noise (SD of the nodule attenuation). The SNR was defined as the ratio of the mean attenuation of the targeted nodule divided by the SD of the nodule. This measurement was made by a single radiologist (T.Y.) ten times for the two nodules on each scan. Different scan series were set side-by-side on the screen and the ROI locations were carefully monitored for measuring comparable points.

To investigate the effects of AIDR3D on image sharpness, on CT scans with the lung window setting (FC52), profile curves of CT density that intersected peripheral pulmonary vessels were drawn and the maximum CT densities of these curves were measured (Figure S2). If a peripheral vessel was not clearly depicted on a scan, it was predicted that the maximum density of the profile curve would be decreased. Ten imitation vessels at different locations were selected for this measurement, and the location of each vessel was captured and recorded for reproducing measured points on different scan series. In addition, density SD of surrounding air (inside the imitated pleural cavity) was measured to clarify the effect of image noise on vessel density. Between scans with and without AIDR3D, differences in maximum vessel density and in image noise of the surrounding air were calculated. The entire process was performed by the same observer (T.Y.).

### Statistical analysis

For qualitative and quantitative measurements of human subjects, statistical comparisons among scans were made by Scheffe's test. Associations between body weight and quantitative image noise were assessed by Spearman rank correlation analysis. Weighted kappa analyses were used to determine inter-observer agreements for the qualitative evaluations of image quality. Inter-observer agreement was considered as minimal (κ<0.21), fair (κ = 0.21–0.40), moderate (κ = 0.41–0.60), substantial (κ = 0.61–0.80), or nearly perfect (κ = 0.81–1.00) [30]. For the phantom study, comparisons of measurements made between scans with and without AIDR3D at each of three tube current settings were made using a Wilcoxon matched-pair signed-rank test. An association between differences in vessel density and those in image noise of surrounding air was assessed by Spearman rank correlation analysis. A *p*-value of<0.05 was considered significant. Statistical analyses were performed by using JMP 8.0 software (SAS Institute, Cary, NC) or Excel Statistics 1.0 software (SSRI Ltd, Tokyo).

## Results

### Radiation dose assessment

Two CTDI vol settings were used in this study based on the patient's body habitus: either 8.6, 4.3, and 2.2 mGy for, respectively, 240, 120, and 60 mA (n = 62; those with a small or medium body habitus) or 9.5, 4.7, and 2.4 mGy (n = 26, large habitus). Mean DLP values for 240, 120, and 60 mA were 328.6, 161.0, and 81.4 mGy⋅cm, respectively. The mean effective radiation dose for the entire protocol used in this study was 8.28. ± 0.95 mSv (range: 5.89 - 10.25 mSv).

### Qualitative image analysis

The results for inter-observer agreement for qualitative image quality with lung and mediastinal window settings are given in Table 1. These results indicated that all inter-observer agreements ranged from moderate to substantial agreement (κ ≥ 0.50).

**Table 1:** Interobserver reproducibility (κ score) among three observers for qualitative assessment*.

	Observer A and B	Observer B and C	Observer A and C
**Lung parenchyma** (FC52, n = 88)			
Upper zone	0.69	0.81	0.67
Middle zone	0.66	0.72	0.67
Lower zone	0.71	0.77	0.71
**Lung disease** (FC52)			
Nodule/mass (n = 25)	0.54	0.65	0.62
Emphysema (n = 18)	0.62	0.54	0.62
Bronchiolitis (n = 12)	0.62	0.59	0.56
Diffuse lung disease (n = 18)	0.69	0.66	0.62
**Mediastinum** (FC13, n = 88)			
Visibility of small structures	0.63	0.63	0.76
Streak artifacts	0.69	0.60	0.69
Shoulder artifacts	0.50	0.66	0.77

* The scale for κ values was as follows: 0.21–0.40, fair agreement; 0.41–0.60, moderate agreement; 0.61–0.80, substantial agreement; 0.81–1.00, almost perfect agreement.

Table 2 summarizes the final scores of the qualitative analyses. All comparisons among the six scan series are shown in online-only material (Table S1). At each of the three different tube currents, CT scans with AIDR3D had significantly higher scores for image quality than those without AIDR3D for all lung zones (*p*<0.0001) and all mediastinal measurements (*p*<0.01). For lung diseases, significant quality improvements using AIDR3D were observed for lung nodule/mass at 240 mA (*p*<0.05) and for three lung disease patterns (nodule/mass, emphysema, and diffuse lung disease) at 120 and 60 mA (at 120 mA, *p*<0.01; at 60 mA, *p*<0.05). At each tube current, although the score for bronchiolitis was higher on scans with AIDR3D than on those without AIDR3D, the differences in these scores were not significant. In each of three scan series with or without AIDR3D, a higher tube current resulted in higher scores. Thus, scans with AIDR3D at 240 mA achieved the highest scores for all measurements.

**Table 2:** Results of qualitative scores* for image quality at different tube currents with AIDR3D and FBP (without AIDR3D).

	AIDR3D			FBP		
	240 mA	120 mA	60 mA	240 mA	120 mA	60 mA
	(84 mAs)	(42 mAs)	(21 mAs)	(84 mAs)	(42 mAs)	(21 mAs)
**Lung parenchyma** (FC52, n = 88)						
Upper zone	4.19	3.69	3.25	2.25	1.64	1.17
Middle zone	4.66	4.15	3.67	3.4	2.89	2.24
Lower zone	4.59	4.07	3.51	3.22	2.47	1.89
**Lung disease** (FC52)						
Nodule/mass (n = 25)	4.28	3.88	3.36	3.32	2.48	2.16
Emphysema (n = 18)**	4	3.33	2.78	3.06	2.22	1.72
Bronchiolitis (n = 12)	4.33	3.92	3.5	3.92	3	2.25
Diffuse lung disease(n = 18)**	4.11	3.44	2.89	3.06	2.22	1.72
**Mediastinum** (FC13, n = 88)						
Visibility of small structures	4.38	3.5	2.69	3.44	2.48	1.44
Streak artifacts	4.07	3.64	3.08	2.9	2.13	1.27
Shoulder artifacts	4.01	2.89	1.77	2.81	1.52	1

* A 5-point scale is used to evaluate image quality: 1, nondiagnostic; 2, poor; 3, acceptable; 4, good; 5, excellent.

** Same scores for scans without AIDR3D (emphysema and diffuse lung disease) were not an error and double-checked by the authors.

**Figure 1:**
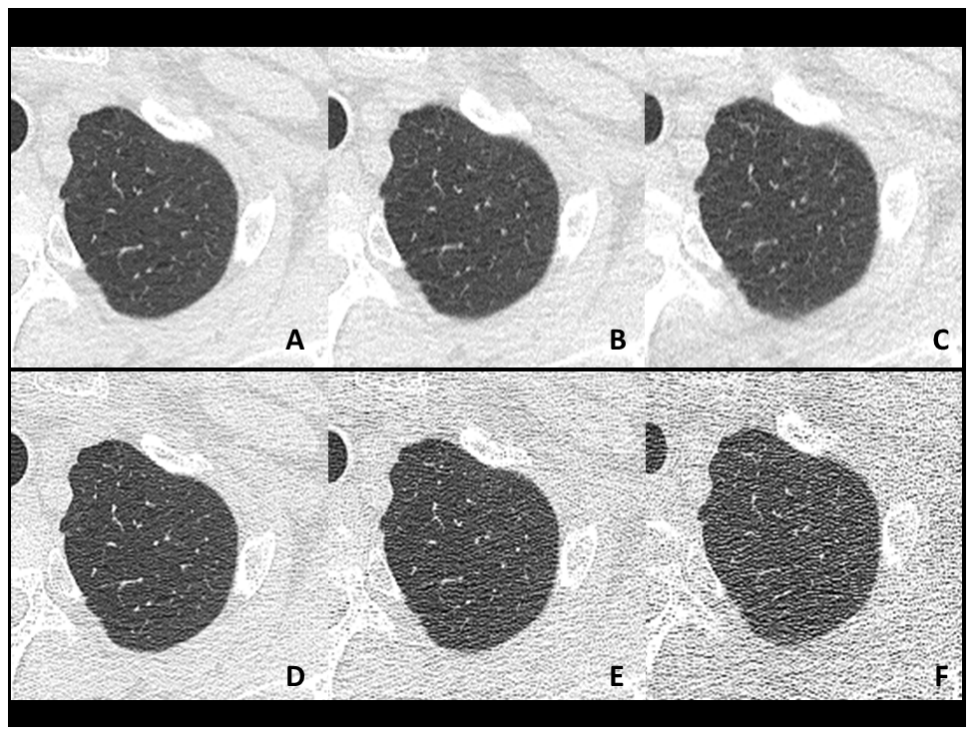
Axial plain chest CT images at the upper lung zone (60-year-old male weighing 76 kg). These images were created from scan data at 240 mA (**A, D**), 120 mA (**B, E**) and 60 mA (**C, F**). The three upper images (**A–C**) were reconstructed using AIDR3D and the three lower images (**D–F**) were reconstructed using a conventional reconstruction mode (Boost3D). Each image pair at the same tube current was created from single row data. Image noise was obviously reduced on images with AIDR3D, particularly at lower tube currents.

**Figure 2:**
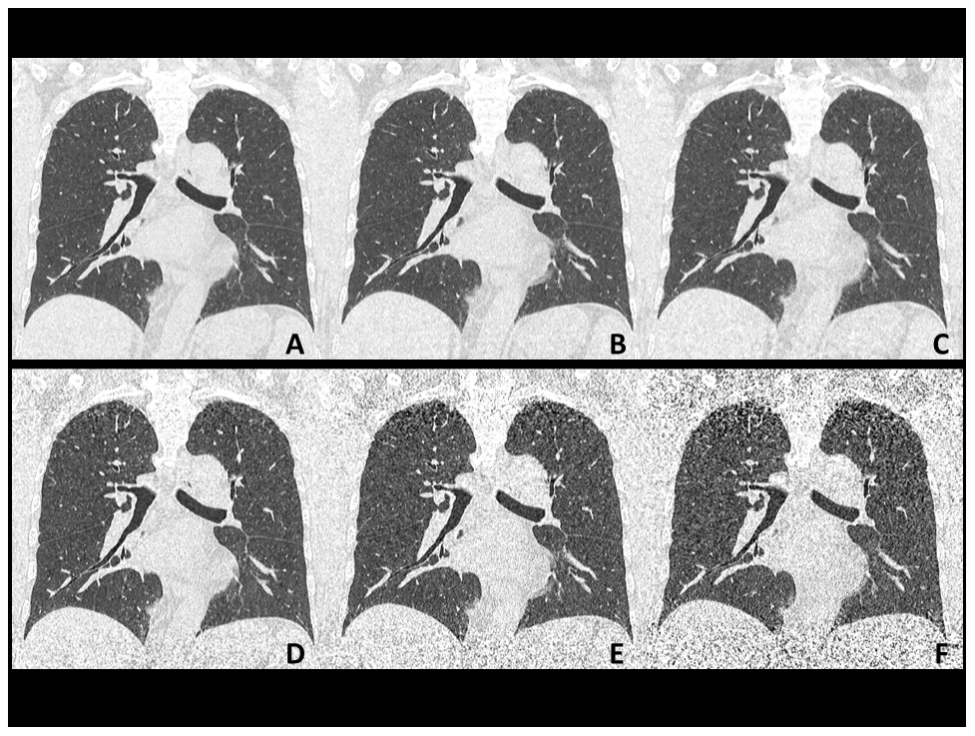
Reconstructed coronal plain chest CT images (56-year-old male weighing 62 kg). Images are arranged as in Figure 1 (**A–C**, with AIDR3D; **D–F**, without AIDR3D; **A** and **D**, at 240 mA; **B** and **E**, at 120 mA; **C** and **F**, at 60 mA). Severe image noise was observed at the upper lung zones and bottoms at 120 and 60 mA without AIDR3D (**E** and **F**), which was obviously improved using AIDR3D (**B** and **C**).

**Figure 3:**
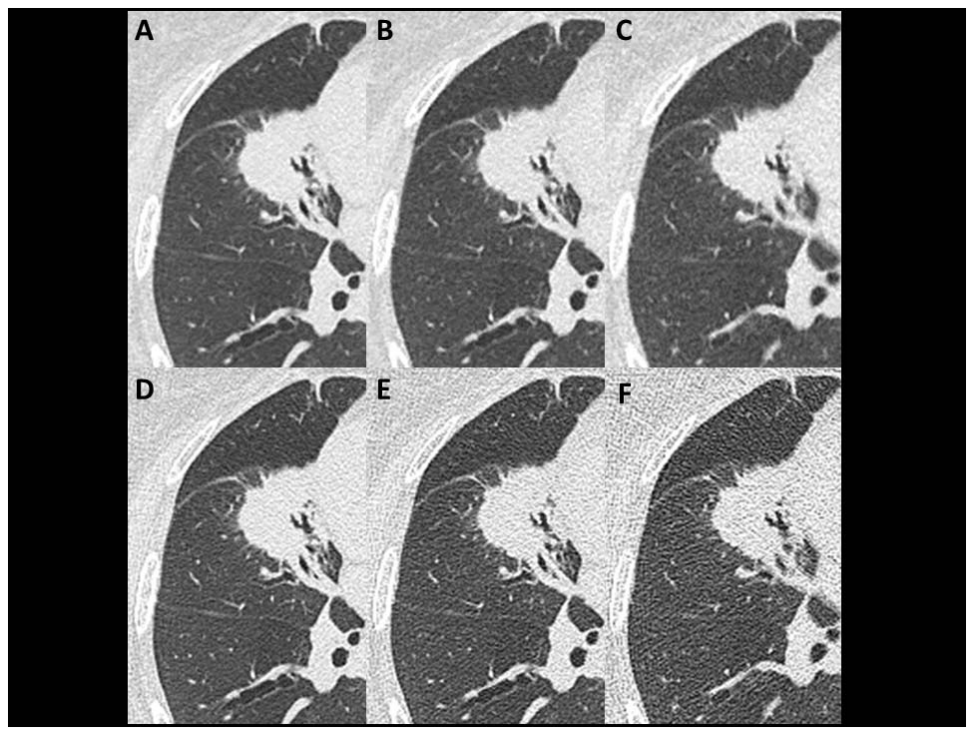
Axial plain chest CT images with a solid lung mass in the right middle lobe (75-year-old female weighing 56 kg). Images are arranged as in Figures 1 and 2. Spiculae were found on all images, while density heterogeneity inside the mass was severe on images at 60 mA without AIDR3D (**F**).

**Figure 4:**
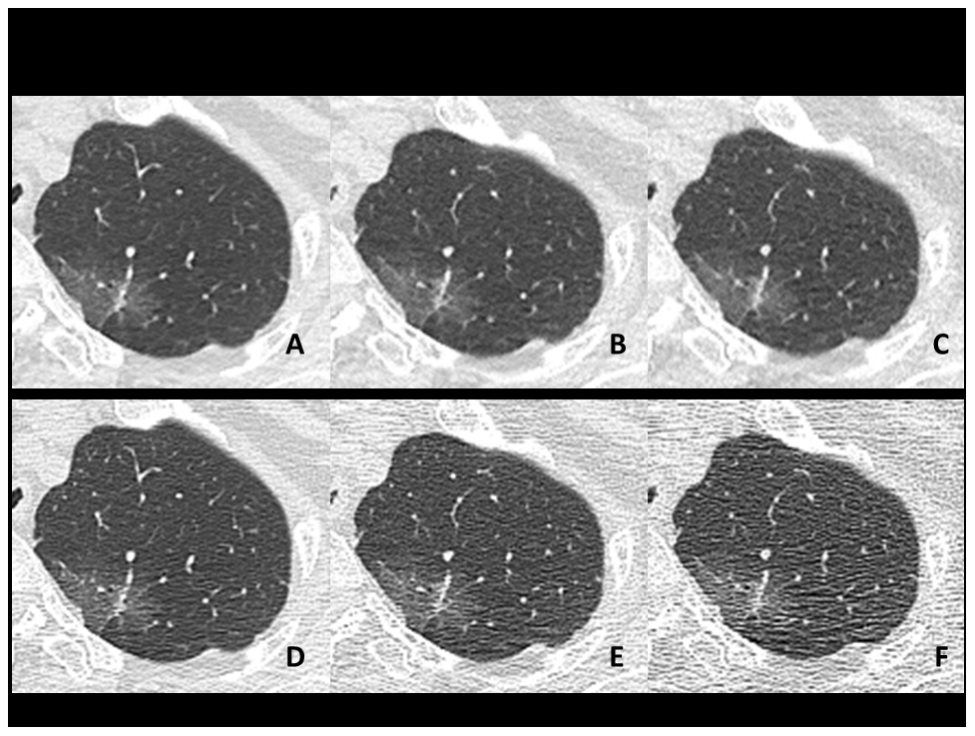
Axial plain chest CT images showing a ground-glass opacity (GGO) nodule in the left apex (74-year-old female weighing 49 kg). Images are arranged as in Figures 1 and 2. Nearly homogeneous density of the nodule was accurately depicted on images with AIDR3D at any of three tube currents (**A–C**). However, on images without AIDR3D (**D–F)**, artificial density heterogeneity due to image noise increased as the tube current decreased from 240 (**D**) to 60 mA (**F**).

**Figure 5:**
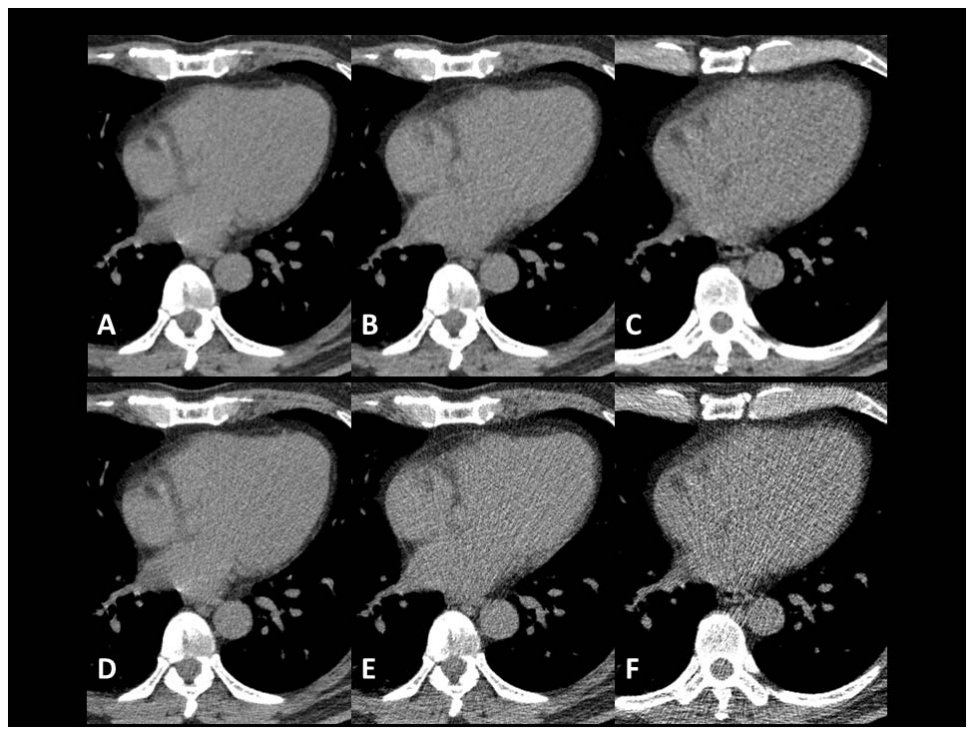
Axial plain chest CT images with a mediastinal setting to assess streak artifacts (55-year-old male weighing 64 kg). A , at 240 mA with AIDR3D; **B**, at 120 mA with AIDR3D; **C**, at 60 mA with AIDR3D; **D**, at 240 mA without AIDR3D; **E**, at 120 mA without AIDR3D; **F**, at 60 mA without AIDR3D. Many radial streaks from the spine were apparent in the heart, particularly on the image without AIDR3D at 60 mA (**F**). These streaks were greatly reduced using AIDR3D (**C**, at 60 mA).

The scores for CT scans with AIDR3D at 120 mA were significantly higher than those without AIDR3D at 240 mA for all lung zones and mediastinal streak artifacts (*p*<0.0001), and slightly higher or equivalent for lung diseases and other mediastinal measurements. Similarly, CT scans with AIDR3D at 60 mA were judged to be superior (all lung zones and mediastinal streak artifacts, *p*<0.0001; lung nodule/mass, *p*<0.05) or equivalent to those without AIDR3D at 120 mA. Example images for the lung parenchyma, lung diseases, and mediastinal measurements are shown in Figures 1, 2, 3, 4, 5 and Figures S3–S7.

### Quantitative image analysis

Table 3 summarizes objective image noise for lung and mediastinal windows. Comparisons among the six scans are given in online-only material (Table S2). At each of the three different tube currents, CT scans with AIDR3D had significantly less image noise than those without AIDR3D for both the lung parenchyma and aorta (*p*<0.0001). In each scan series with and without AIDR3D, a higher tube current resulted in less image noise. Therefore, similar to the qualitative assessments, a CT scan with AIDR3D at 240 mA had minimum image noise in all measurements. Further, at all measured points, image noise on CT scans with AIDR3D at 120 mA was significantly less than that without AIDR3D at 240 mA (*p*<0.0001). Also, image noise on CT scans with AIDR3D at 60 mA was significantly less than that without AIDR3D at 120 mA (*p*<0.0001).

**Table 3:** Results of quantitative image noise (SD measurement*) at different tube currents with AIDR3D and FBP (without AIDR3D).

	AIDR3D			FBP		
	240 mA	120 mA	60 mA	240 mA	120 mA	60 mA
	(84 mAs)	(42 mAs)	(21 mAs)	(84 mAs)	(42 mAs)	(21 mAs)
**Lung parenchyma** (FC52)						
Apex	106.8 ± 20.8	112.7 ± 20.1	115.7 ± 20.3	216.8 ± 33.9	297.1 ± 55.2	423.9 ± 87.1
Upper middle (at the carina)	80.4 ± 18.1	87.2 ± 17.5	94.6 ± 16.8	136.3 ± 32.0	171.5 ± 51.0	231.4 ± 83.7
Lower middle (at the LPV)	78.9 ± 16.0	86.2 ± 15.3	94.2 ± 14.2	131.1 ± 29.8	164.8 ± 45.8	227.8 ± 80.2
Bottom	107.8 ± 25.7	116.5 ± 25.0	120.9 ± 24.7	172.7 ± 39.3	219.1 ± 57.0	302.2 ± 99.5
**Mediastinum** - **Aorta** (FC13)						
Aortic arch	10.8 ± 1.9	13.1 ± 2.3	16.3 ± 3.6	18.4 ± 5.9	26.1 ± 10.1	37.9 ± 15.2
Descending aorta	12.4 ± 1.7	15.0 ± 2.5	18.2 ± 2.9	21.7 ± 5.3	30.9 ± 9.6	45.9 ± 15.1

*Definition of abbreviations*: SD: standard deviation; LPV: lower pulmonary vein.

* The standard deviation of the CT values with a fixed circular region of interest (10 mm in radius) was measured.

There were significant correlations between body weight and image noise for the lung parenchyma (FC52) on all series without AIDR3D (*p*<0.05) (Table 4). Correlation coefficients increased as the tube current decreased from 240 to 60 mA. These correlations were not observed on any series for a lung window with AIDR3D. In contrast, with the mediastinal setting (FC13), significant correlations were found between body weight and image noise on all scans with and without AIDR3D. In general, correlation coefficients were higher for scans without AIDR3D than for those with AIDR3D.

**Table 4:** Correlations between quantitative image noise and body weight*.

	AIDR3D			FBP		
	240 mA	120 mA	60 mA	240 mA	120 mA	60 mA
	(84 mAs)	(42 mAs)	(21 mAs)	(84 mAs)	(42 mAs)	(21 mAs)
**Lung parenchyma** (FC52)						
Apex	0.04	0.01	−0.04	0.48	0.58	0.60
	(NS)	(NS)	(NS)	(*p*<0.0001)	(*p*<0.0001)	(*p*<0.0001)
Upper middle (at the carina)	0.02	0.02	0.00	0.27	0.35	0.41
	(NS)	(NS)	(NS)	(*p*<0.05)	(*p*<0.001)	(*p*<0.0001)
Lower middle (at the LPV)	0.13	0.13	0.03	0.42	0.47	0.48
	(NS)	(NS)	(NS)	(*p*<0.0001)	(*p*<0.0001)	(*p*<0.0001)
Bottom	−0.05	−0.12	−0.16	0.36	0.48	0.56
	(NS)	(NS)	(NS)	(*p*<0.001)	(*p*<0.0001)	(*p*<0.0001)
**Mediastinum - Aorta** (FC13)						
Aortic arch	0.61	0.63	0.59	0.65	0.69	0.66
	(*p*<0.0001)	(*p*<0.0001)	(*p*<0.0001)	(*p*<0.0001)	(*p*<0.0001)	(*p*<0.0001)
Descending aorta	0.38	0.41	0.36	0.64	0.65	0.68
	(*p*<0.001)	(*p*<0.0001)	(*p*<0.001)	(*p*<0.0001)	(*p*<0.0001)	(*p*<0.0001)

*Definition of abbreviations*: LPV: lower pulmonary vein; NS: not significant.

* Spearman rank correlation analysis was used to evaluate correlations between image noise and body weight. Correlation coefficient (ρ) and p values are shown.

### Phantom study

Table 5 shows results of our phantom study. At each of the three tube current settings, the SNRs of imitation lung nodules were greater (higher for the solid nodule with positive CT density, and lower for the GGO nodule with negative density) on scans with AIDR3D than on those without AIDR3D (*p*<0.01). For both solid and GGO nodules, SNR measurements approached zero when the tube current decreased from 240 to 60 mA, even when using AIDR3D, which suggested that effective signals created by the lung nodules were interfered with to a greater extent by image noise at lower tube current settings.

**Table 5:** Results of phantom study at different tube currents with AIDR3D and FBP (without AIDR3D).

	AIDR3D			FBP		
	240 mA	120 mA	60 mA	240 mA	120 mA	60 mA
	(84 mAs)	(42 mAs)	(21 mAs)	(84 mAs)	(42 mAs)	(21 mAs)
**lung nodule SNR** (FC13)						
Solid	10.4 ± 1.1	8.8 ± 0.8	7.8 ± 1.4	7.7 ± 0.8	5.8 ± 0.3	4.4 ± 0.3
Ground-glass opacity	− 78.2 ± 13.8	−59.2 ± 8.9	−50.4 ± 4.0	−51.5 ± 4.8	−37.8 ± 5.1	−28.7 ± 1.8
**Image sharpness** (FC52)						
Vessel density (HU)	−325.5 ± 114.8	−340.8 ± 155.7	−437.4 ± 168.0	−179.8 ± 118.3	−94.5 ± 151.9	−145.9 ± 116.5
Image noise (HU)	40.4 ± 6.4	50.5 ± 5.6	58.2 ± 6.3	92.0 ± 19.4	129.5 ± 26.5	164.5 ± 38.8

*Definition of abbreviations*: SNR: signal-to-noise ratio; HU: Hounsfield Unit.

For the density measurements of peripheral pulmonary vessels, vascular density decreased from 240 to 60 mA when using AIDR3D. Further, at each of the three tube current settings, scans without AIDR3D gave higher densities than scans with AIDR3D (*p*<0.01). However, the differences in vessel density between scans with and without AIDR3D were positively correlated with the differences in image noise of background air between these scans (ρ = 0.623, *p*<0.001) (Figure S8). These observations implied that small structures, such as peripheral vessels or bronchial walls, may be depicted more brightly on scans without AIDR3D. However, this apparent brightness or sharpness on scans without AIDR3D could have been artificially caused by image noise overlying these structures (Figure S9).

## Discussion

In this prospective study using scan data at three tube current settings, we demonstrated that the AIDR3D mode improved image quality either significantly or insignificantly at each of three different tube currents used. With both lung and mediastinal settings, 240 mA with AIDR3D provided the highest image quality among the six scan series. Further, CT scan images with AIDR3D at 120 mA were superior or equivalent to those without AIDR3D at 240 mA, which suggested that CT scans using AIDR3D at 120 mA would be more practical for clinical use than those using a conventional reconstruction mode at 240 mA. Similarly, scan images with AIDR3D at 60 mA were superior or equivalent to those without AIDR3D at 120 mA. These observations suggest that using AIDR3D could potentially reduce radiation exposure for clinical chest CT imaging by at least 50%.

### Clinical applications of AIDR3D for chest CT

It was recently reported that the AIDR3D mode achieved radiation dose reduction of 64.2% for chest CT with automatic exposure control (AEC) [26] and a reduction of 50% for coronary CT angiography [22]. Similar results were shown in recent studies using different iterative reconstruction techniques [16], [19]. In this study, we also demonstrated that by using AIDR3D, CT scans using 50% of the radiation dose provided superior or equivalent images as compared to those using full doses and without AIDR3D. Based on these observations, the advantages of AIDR3D included both dose reductions and improved image quality with respect to several points.

First, the positive effects of AIDR3D were more obvious for scans made with lower tube currents. Objective measurements demonstrated that when not using AIDR3D, image noise at each point increased rapidly from 240 to 60 mA, and image noise at 60 mA was approximately twice the level of noise at 240 mA. This rapid increase in image noise was not observed when using AIDR3D, although image noise gradually increased with a dose decrease from 240 to 60 mA. Thus, the greatest improvement in image noise between scans with and without AIDR3D was found at 60 mA. Greater improvements in image quality at lower tube currents would enable greater dose reductions for chest CT.

Second, AIDR3D contributed to minimizing the influence of body habitus on image quality. Particularly for patients with large body weights, effective x-ray signals are relatively insufficient due to photon absorption, even when using a regular tube current setting, which results in increased image noise. Similar to the positive effect of AIDR3D at lower tube currents, AIDR3D compensated for these disadvantages of larger patients and improved image quality. This would partially explain the non-significant correlations between patient body weight and image noise when using the lung window setting in this study (Table 4). However, if effective signals are absolutely insufficient due to inappropriate photon counts and the signal-to-noise ratio is very low when creating CT images, AIDR3D cannot compensate for the signal defects, which results in unclear, melded areas that are typically observed around the shoulder joints (Figure S7). Thus, optimal tube currents for large patients, including AEC adjustments, should be considered carefully for scans with AIDR3D.

Third, for lung parenchyma, the upper parts of lungs generally had considerable noise due to artifacts from shoulder joints on scans without AIDR3D, which was shown by lower subjective scores at the upper lung zone than those at other lung zones, and higher objective noise at the apex than at middle lung zones. This was dramatically improved when using AIDR3D, and subjective scores of the upper lung zone were slightly worse than those of the middle zones.

Based on these advantages of AIDR3D in terms of dose reduction and image quality improvement compared to a conventional reconstruction method, it is strongly recommended that the AIDR3D method is used for clinical chest CT.

### Image noise reduction and decrease in sharpness

Several studies have shown that there is a trade-off between noise reduction and image sharpness on clinical CT images [31]–[33]. Because the AIDR3D method can significantly reduce image noise, it would be expected that image sharpness would be affected to some extent, particularly at the lung window setting. In fact, this phenomenon might have been demonstrated by the differences in the improvements of objective scores between lung parenchyma and lung diseases; overall, AIDR3D had a greater beneficial effect on scores for lung parenchyma than on scores for lung diseases. This observation implies that image quality of abnormal CT findings in the lung field does not simply reflect image noise, which has been rarely discussed in the previous literatures.

In some cases, the observers felt that images with AIDR3D were a little unclear for depicting small structures of lung diseases, which resulted in scores identical to those without AIDR3D, particularly for bronchiolitis. These observations were partly supported by our observations with a phantom study (Figure S9). When using AIDR3D, the maximum densities of imitated peripheral vessels decreased as the tube current setting decreased from 240 to 60 mA, and larger disturbances in vascular densities were observed at lower tube current settings between when using and not using AIDR3D. Thus, it would be expected that small structures would be more sharply or brightly depicted on scans without AIDR3D than on those with AIDR3D; however, these differences in vascular density were correlated with the differences in background image noise, which may prevent observers from interpreting the true characteristics of these structures. Further, in clinical use, image sharpness might be guaranteed by using manufacturer recommendations to adjust reconstruction kernels to sharper ones for AIDR3D (for example, changing kernels from FC52 to FC53). The reconstruction kernel was not modified in this study between scans with and without AIDR3D, because our primary goal was to examine the direct effects of the AIDR3D mode.

Further, the positive effect of AIDR3D for noise or artifact reduction was obviously shown in some patient groups with homogenous lung lesions. As shown in Figure 4, GGO nodule/mass with a homogeneous CT density was often depicted as heterogeneous structures on scans without AIDR3D due to image noise or artifact. This artificial, apparent density heterogeneity was resolved by using AIDR3D at each tube current. Similar observations were found in patients with diffuse lung disease, which included multiple GGO lesions, or patients exhibiting large consolidations. It is beneficial for clinical diagnosis to evaluate whether or not lung lesions are truly homogeneous or heterogeneous on images with minimum noise, which would be possible using AIDR3D. We currently believe that the benefit of AIDR3D for noise reduction outweighs a slight decrease in image sharpness, which could be easily adjusted using sharper reconstruction kernels.

This study had several limitations. First, image quality for the lung parenchyma and pulmonary diseases was evaluated using an overall score. Thus, particularly for lung diseases, image quality based on more specific CT findings, such as visibility of interlobular septal thickening or peripheral bronchiectasis, was not evaluated in this study. Although the major aim of this study was to investigate the effects of AIDR3D for general chest CT imaging, we might have obtained different results if we had used a different scoring system for more specific CT findings. Second, although this study was conducted as a multicenter trial, the number of patients was relatively small, particularly the numbers of patients with lung diseases (12 to 25 patients). Although we believe that the measurements obtained were not artificially skewed or exaggerated due to the small number of patients, more detailed studies with greater numbers of patients are recommended to investigate the effects of AIDR3D for each type of lung disease. Third, we did not use AEC in this study, although it is frequently used in routine clinical practice. This was because one of the original aims of this study was to investigate the relationship between body weight and image quality with fixed tube current settings. However, AEC is very powerful for achieving consistent image quality among patients with varying body habitus. When combining AEC and AIDR3D, a larger reduction in radiation dose might be feasible without degradation on chest CT images. Fourth, we did not arrive at a definitive conclusion for recommending a minimum tube current setting with AIDR3D. Because all qualitative assessments were made on CT scans that were completely randomized, the observers did not compare scans for the same patients with different tube currents or with/without AIDR3D, which did not allow for comparisons for the visibility of small structures or abnormal findings in the lungs side-by-side. Fifth, we only evaluated an iterative reconstruction method developed by a single manufacturer. More studies will be needed to compare the advantages of iterative reconstruction techniques from multiple manufacturers for future clinical diagnosis and lung screening. Sixth, image sharpness was expressed as the highest attenuation of the imitation vessels in the phantom study. Although we could not find a similar previous research regarding quantitatively measured image sharpness of clinical chest CT, this method might have been replaced by better approaches, such as line profiles for vascular density.

In conclusion, the AIDR3D method provides better chest CT image quality at standard and reduced tube current settings. The positive effects of AIDR3D can compensate for the effects of a 50% dose reduction, and a decrease in the radiation dose should be feasible by using AIDR3D.

## Supporting Information

Figure S1 Axial and reconstructed coronal CT images of a chest phantom (N1 Lungman). In the right pleural cavity, an imitation solid nodule was inserted (arrow). In the left cavity, an imitation GGO nodule was placed (arrowhead). Note that a reconstructed coronal image was not used for image analysis, used as a reference for this paper.
(TIF)

Figure S2 Example measurement for an imitated pulmonary vessel. A profile curve of CT density that was obtained from a crossing line (shown in yellow) is shown. With this measurement, the maximum density of the measured vessel was -19.0 HU. On the same image, image noise (density SD) of background air was measured as 32.3 HU (circular region of interest indicated by a pink line).
(TIF)

Figure S3 Axial plain chest CT images for a subject with pulmonary emphysema (65-year-old male weighing 70 kg). **A-C**, with AIDR3D; **D-F**, without AIDR3D; **A** and **D**, at 240 mA; **B** and **E**, at 120 mA; **C** and **F**, at 60 mA. Boundaries of emphysematous spaces were not clearly visualized on the image without AIDR3D at 60 mA (**F**). This was improved using AIDR3D (**C**, at 60 mA); however, the contrast between emphysema and relatively normal parenchyma was still weak as compared with images at 240 mA (**A**, with AIDR3D; **D**, without AIDR3D).
(TIF)

Figure S4 Axial plain chest CT images demonstrating bronchiolitis (78-year-old male weighing 56 kg). Images are arranged as in Figure E1. Multiple centrilobular nodules were observed in the right upper lobe (ovoid circle, **A**). Some nodules and small pulmonary vessels, particularly in the dorsal part of the lung, were not clearly depicted on the image without AIDR3D at 60 mA due to image artifact (**F**). Although this was partially restored using AIDR3D (**C**, at 60 mA), sharpness and contrast of these small structures was less clear than on images at 240 mA (**A**, with AIDR3D; **D**, without AIDR3D).
(TIF)

Figure S5 Axial plain chest CT images for diffuse lung disease (73-year-old female weighing 62 kg). Images are arranged as in Figure E1. Multiple GGO and reticular shadows, accompanying slight bronchiectasis, were observed in the right lower lobe. Density graduations of GGO lesions were more easily evaluated on images with AIDR3D (**A-C**). On images without AIDR3D at 120 (**E**) and 60 mA (**F**), less affected lung parenchyma also appeared heterogeneous due to image noise and artifact.
(TIF)

Figure S6 Axial plain chest CT images with a mediastinal setting to evaluate visibility of small structures inside the mediastinum (67-year-old male weighing 81 kg). Images are arranged as in previous figures. Small mediastinal lymph nodes and vessels were depicted on the image with AIDR3D at 240 mA (arrows, **A**). Some of these were difficult to be pointed out due to multiple linear artifacts on the image without AIDR3D at 60 mA (**F**).
(TIF)

Figure S7 Axial plain chest CT images with a mediastinal setting to evaluate shoulder artifacts (71-year-old female weighing 65 kg). Images are arranged as in previous figures. On the image without AIDR3D at 60 mA (**F**), multiple linear artifacts formed a horizontal density layer (arrows) in the dorsal part of the chest wall, which were less frequently observed on images at higher currents. These artifacts were reduced using AIDR3D (**C**, at 60 mA); however, insufficient photon counts due to a beam hardening effect resulted in an artificial black zone (ovoid circle, **C**).
(TIF)

Figure S8 Correlation between differences in maximum vascular density and those in background image noise. These differences were obtained by comparing scans with and without AIDR3D at each tube current setting. Plots shown by open circles were obtained from scans at 240 mA, by closed (black) circles at 120 mA, and by open boxes at 60 mA. There was a significant correlation between the differences in maximum vascular density and those in background image noise (ρ = 0.623, *p*<0.001).
(TIF)

Figure S9 Effect of image noise on vascular density. Demonstration phantom images were created from a single row of data using 120 mA (**A**, without AIDR3D; **B**, with AIDR3D). Both images are shown with a fixed window setting (level: -600 HU; width: 1600 HU). An imitated peripheral vessel (arrows) was more brightly depicted on the image without AIDR3D (**A**) than that with AIDR3D (**B**). However, this apparent brightness on the image without AIDR3D seemed to be caused by more severe image noise shown in the background (See rectangle on **A**), which was clearly reduced on the image with AIDR3D (rectangle on **B**). Similar phenomena were frequently observed throughout the images, which may lead to the impression that image sharpness is slightly better on an image without AIDR3D (**A**) than on one with AIDR3D (**B**).
(TIF)

Table S1 Comparison of subjective scores for image quality among six scan series.
(DOCX)

Table S2 Comparison of objective image noise among six scan series.
(DOCX)
