# Supplementary figures and images for: Adaptive Iterative Dose Reduction Using Three Dimensional Processing (AIDR3D) Improves Chest CT Image Quality and Reduces Radiation Exposure
Source: PLoS One. 2014 Aug 25;9(8):e105735. doi: 10.1371/journal.pone.0105735 (PMC4143266; doi:10.1371/journal.pone.0105735)

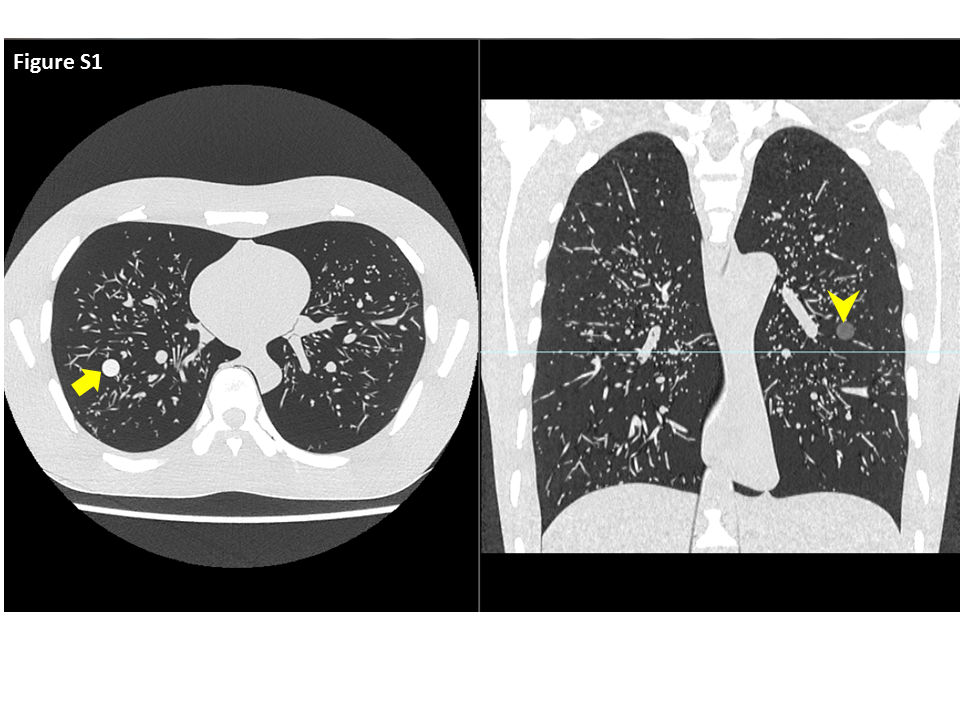

Supplement: Figure S1 — Axial and reconstructed coronal CT images of a chest phantom (N1 Lungman). In the right pleural cavity, an imitation solid nodule was inserted (arrow). In the left cavity, an imitation GGO nodule was placed (arrowhead). Note that a reconstructed coronal image was not used for image analysis, used as a reference for this paper. (TIF) [file pone.0105735.s001.tif]

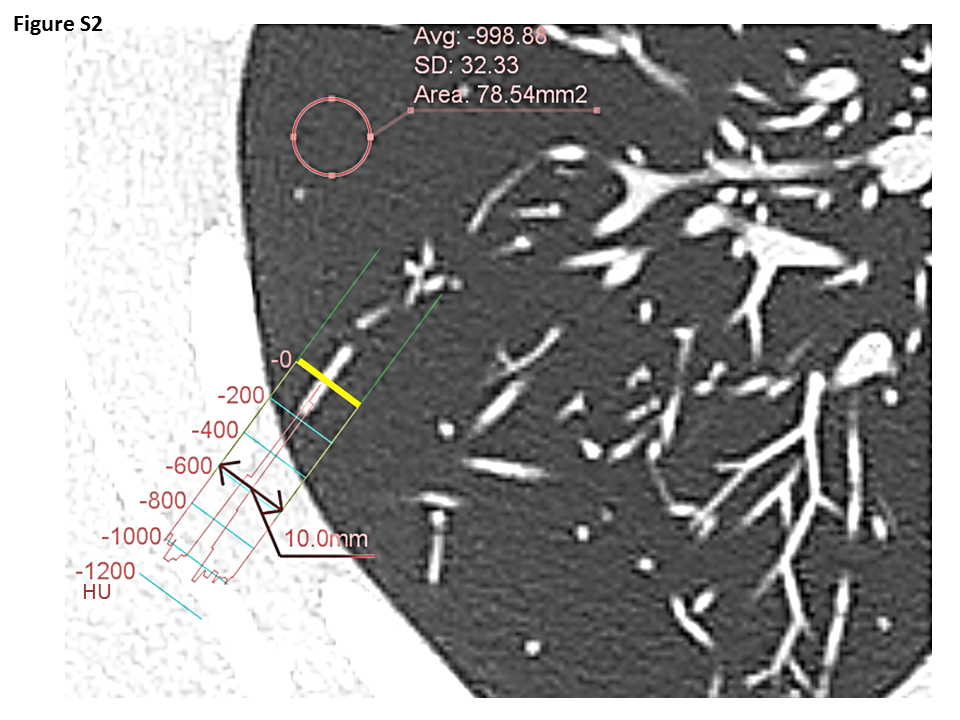

Supplement: Figure S2 — Example measurement for an imitated pulmonary vessel. A profile curve of CT density that was obtained from a crossing line (shown in yellow) is shown. With this measurement, the maximum density of the measured vessel was -19.0 HU. On the same image, image noise (density SD) of background air was measured as 32.3 HU (circular region of interest indicated by a pink line). (TIF) [file pone.0105735.s002.tif]

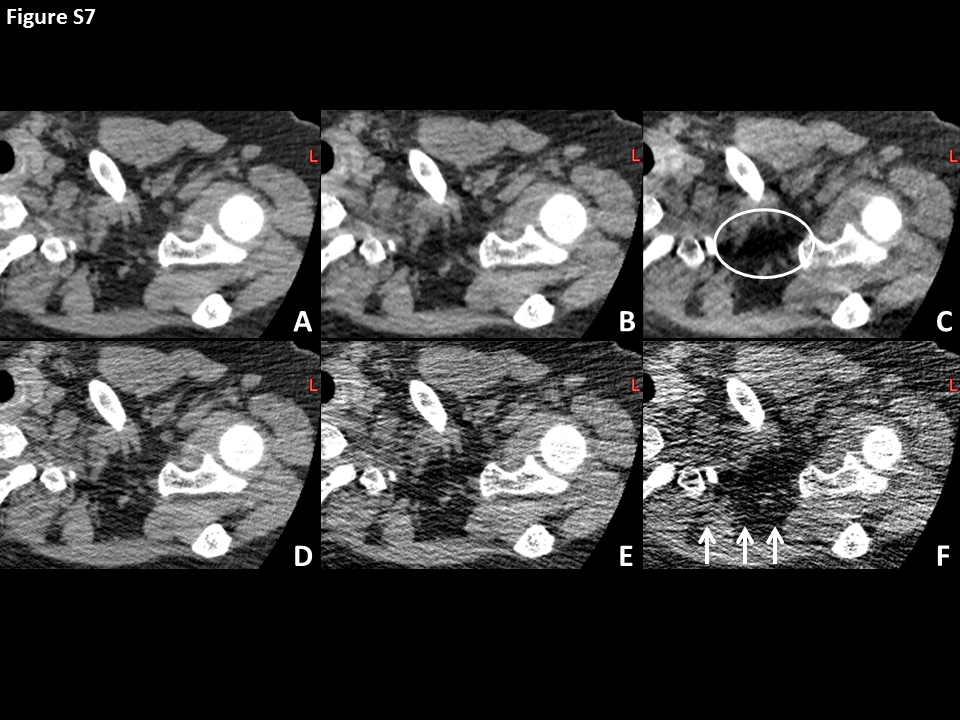

Supplement: Figure S7 — Axial plain chest CT images with a mediastinal setting to evaluate shoulder artifacts (71-year-old female weighing 65 kg). Images are arranged as in previous figures. On the image without AIDR3D at 60 mA (F), multiple linear artifacts formed a horizontal density layer (arrows) in the dorsal part of the chest wall, which were less frequently observed on images at higher currents. These artifacts were reduced using AIDR3D (C, at 60 mA); however, insufficient photon counts due to a beam hardening effect resulted in an artificial black zone (ovoid circle, C). (TIF) [file pone.0105735.s007.tif]

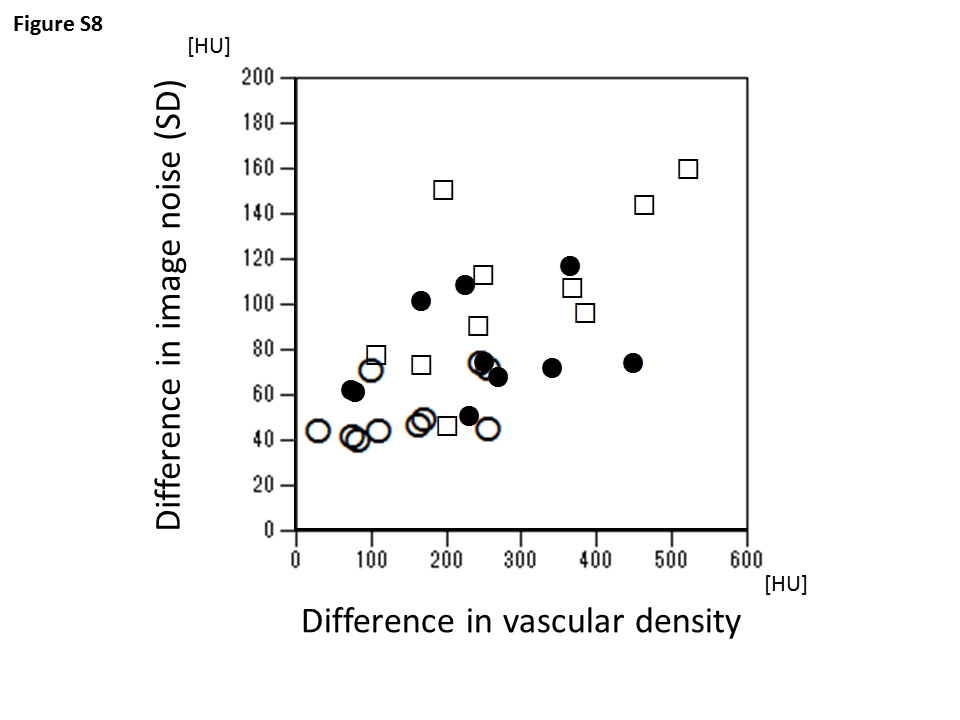

Supplement: Figure S8 — Correlation between differences in maximum vascular density and those in background image noise. These differences were obtained by comparing scans with and without AIDR3D at each tube current setting. Plots shown by open circles were obtained from scans at 240 mA, by closed (black) circles at 120 mA, and by open boxes at 60 mA. There was a significant correlation between the differences in maximum vascular density and those in background image noise (ρ = 0.623, p<0.001). (TIF) [file pone.0105735.s008.tif]

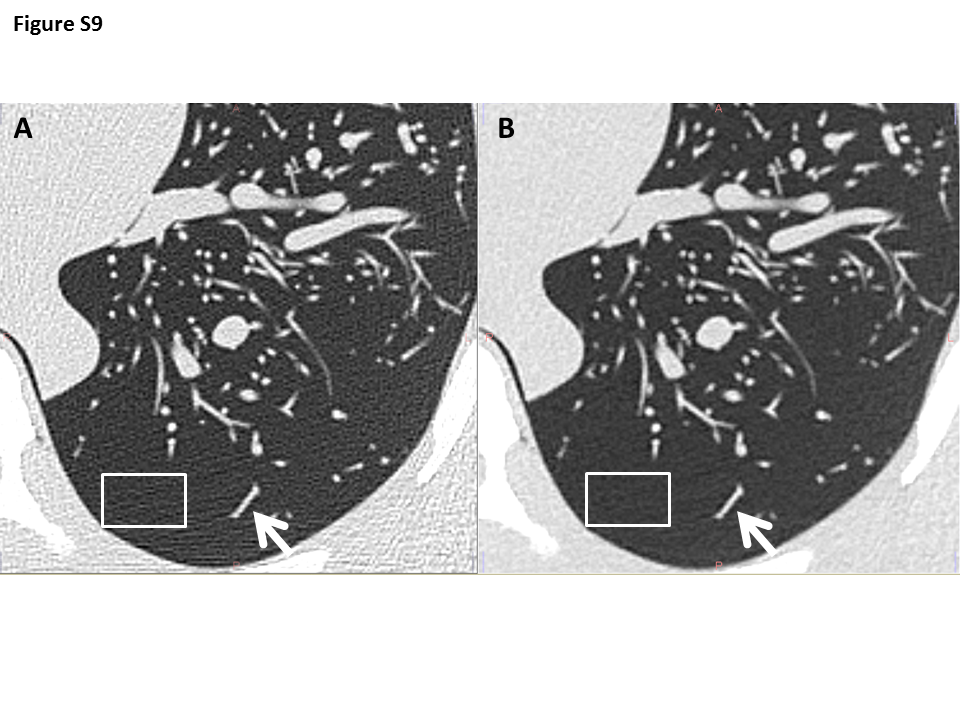

Supplement: Figure S9 — Effect of image noise on vascular density. Demonstration phantom images were created from a single row of data using 120 mA (A, without AIDR3D; B, with AIDR3D). Both images are shown with a fixed window setting (level: -600 HU; width: 1600 HU). An imitated peripheral vessel (arrows) was more brightly depicted on the image without AIDR3D (A) than that with AIDR3D (B). However, this apparent brightness on the image without AIDR3D seemed to be caused by more severe image noise shown in the background (See rectangle on A), which was clearly reduced on the image with AIDR3D (rectangle on B). Similar phenomena were frequently observed throughout the images, which may lead to the impression that image sharpness is slightly better on an image without AIDR3D (A) than on one with AIDR3D (B). (TIF) [file pone.0105735.s009.tif]
