# Supplementary material for: Adaptive Iterative Dose Reduction Using Three Dimensional Processing (AIDR3D) Improves Chest CT Image Quality and Reduces Radiation Exposure
Source: PLoS One. 2014 Aug 25;9(8):e105735. doi: 10.1371/journal.pone.0105735 (PMC4143266; doi:10.1371/journal.pone.0105735)
Supplement: Table S1 — Comparison of subjective scores for image quality among six scan series. (DOCX) [file pone.0105735.s010.docx]

**Table S1** **(Supporting Information)** Comparison of subjective scores for image quality among six scan series

| Pairs |  | **Lung zone** |  |  | | **Lung disease** |  |  |  | **Mediastinum** |  |  |
| --- | --- | --- | --- | --- | --- | --- | --- | --- | --- | --- | --- | --- |
|  |  | upper | middle | lower | | nodule/mass | emphysema | bronchiolitis | DLD | visibility | streak | shoulder |
| 240 AIDR3D | 120 AIDR3D | NS(0.22) | p<0.05 | NS(0.076) | NS(0.84) | | NS(0.63) | NS(0.91) | NS(0.62) | p<0.001 | NS(0.34) | p<0.0001 |
| 240 AIDR3D | 60 AIDR3D | p<0.0001 | p<0.0001 | p<0.0001 | p<0.01 | | p<0.01 | NS(0.19) | p<0.01 | p<0.0001 | p<0.0001 | p<0.0001 |
| 240 AIDR3D | 240 FBP | p<0.0001 | p<0.0001 | p<0.0001 | p<0.01 | | NS(0.1) | NS(0.87) | NS(0.08) | p<0.0001 | p<0.0001 | p<0.0001 |
| 240 AIDR3D | 120 FBP | p<0.0001 | p<0.0001 | p<0.0001 | p<0.0001 | | p<0.0001 | p<0.01 | p<0.0001 | p<0.0001 | p<0.0001 | p<0.0001 |
| 240 AIDR3D | 60 FBP | p<0.0001 | p<0.0001 | p<0.0001 | p<0.0001 | | p<0.0001 | p<0.0001 | p<0.0001 | p<0.0001 | p<0.0001 | p<0.0001 |
| 120 AIDR3D | 60 AIDR3D | NS(0.294) | p<0.05 | p<0.05 | NS(0.30) | | NS(0.52) | NS(0.82) | NS(0.54) | p<0.0001 | p<0.05 | p<0.0001 |
| 120 AIDR3D | 240 FBP | p<0.0001 | p<0.0001 | p<0.0001 | NS(0.30) | | NS(0.92) | NS(1) | NS(0.91) | NS(1) | p<0.0001 | NS(1) |
| 120 AIDR3D | 120 FBP | p<0.0001 | p<0.0001 | p<0.0001 | p<0.0001 | | p<0.01 | NS(0.08) | p<0.01 | p<0.0001 | p<0.0001 | p<0.0001 |
| 120 AIDR3D | 60 FBP | p<0.0001 | p<0.0001 | p<0.0001 | p<0.0001 | | p<0.0001 | p<0.001 | p<0.0001 | p<0.0001 | p<0.0001 | p<0.0001 |
| 60 AIDR3D | 240 FBP | p<0.001 | NS(0.585) | NS(0.69) | NS(1) | | NS(0.98) | NS(0.87) | NS(0.99) | p<0.0001 | NS(0.89) | p<0.0001 |
| 60 AIDR3D | 120 FBP | p<0.0001 | p<0.0001 | p<0.0001 | P<0.05 | | NS(0.47) | NS(0.74) | NS(0.49) | NS(0.85) | p<0.0001 | NS(0.8) |
| 60 AIDR3D | 60 FBP | p<0.0001 | p<0.0001 | p<0.0001 | p<0.01 | | p<0.05 | NS(0.07) | p<0.05 | p<0.0001 | p<0.0001 | p<0.01 |
| 240 FBP | 120 FBP | p<0.05 | p<0.01 | p<0.001 | p<0.05 | | NS(0.11) | NS(0.11) | NS(0.14) | p<0.0001 | p<0.001 | p<0.0001 |
| 240 FBP | 60 FBP | p<0.0001 | p<0.0001 | p<0.0001 | p<0.01 | | p<0.01 | p<0.001 | p<0.01 | p<0.0001 | p<0.0001 | p<0.0001 |
| 120 FBP | 60 FBP | NS(0.284) | p<0.05 | NS(0.10) | NS(0.94) | | NS(0.86) | NS(0.8) | NS(0.89) | p<0.0001 | p<0.05 | NS(0.17) |

*Definition of abbreviation*: DLD: diffuse lung disease
