# Supplementary material for: Adaptive Iterative Dose Reduction Using Three Dimensional Processing (AIDR3D) Improves Chest CT Image Quality and Reduces Radiation Exposure
Source: PLoS One. 2014 Aug 25;9(8):e105735. doi: 10.1371/journal.pone.0105735 (PMC4143266; doi:10.1371/journal.pone.0105735)
Supplement: Table S2 — Comparison of objective image noise among six scan series. (DOCX) [file pone.0105735.s011.docx]

**Table S2 (Supporting Information)** Comparison of objective image noise among six scan series

| Pairs |  | **Lung** (FC52) |  | |  | |  | **Aorta** (FC13) |  |
| --- | --- | --- | --- | --- | --- | --- | --- | --- | --- |
|  |  | Apex | Upper middle | Lower middle | | Bottom | | Aortic arch | Descending aorta |
| 240 AIDR3D | 120 AIDR3D | NS(0.09) | p<0.05 | NS(0.09) | | NS(0.08) | | p<0.05 | p<0.05 |
| 240 AIDR3D | 60 AIDR3D | p<0.01 | p<0.0001 | p<0.0001 | | p<0.001 | | p<0.0001 | p<0.0001 |
| 240 AIDR3D | 240 FBP | p<0.0001 | p<0.0001 | p<0.0001 | | p<0.0001 | | p<0.0001 | p<0.0001 |
| 240 AIDR3D | 120 FBP | p<0.0001 | p<0.0001 | p<0.0001 | | p<0.0001 | | p<0.0001 | p<0.0001 |
| 240 AIDR3D | 60 FBP | p<0.0001 | p<0.0001 | p<0.0001 | | p<0.0001 | | p<0.0001 | p<0.0001 |
| 120 AIDR3D | 60 AIDR3D | NS(0.90) | NS(0.22) | NS(0.16) | | NS(0.64) | | p<0.01 | p<0.01 |
| 120 AIDR3D | 240 FBP | p<0.0001 | p<0.0001 | p<0.0001 | | p<0.0001 | | p<0.0001 | p<0.0001 |
| 120 AIDR3D | 120 FBP | p<0.0001 | p<0.0001 | p<0.0001 | | p<0.0001 | | p<0.0001 | p<0.0001 |
| 120 AIDR3D | 60 FBP | p<0.0001 | p<0.0001 | p<0.0001 | | p<0.0001 | | p<0.0001 | p<0.0001 |
| 60 AIDR3D | 240 FBP | p<0.0001 | p<0.01 | p<0.01 | | p<0.001 | | NS(0.47) | NS(0.16) |
| 60 AIDR3D | 120 FBP | p<0.0001 | p<0.0001 | p<0.0001 | | p<0.0001 | | p<0.0001 | p<0.0001 |
| 60 AIDR3D | 60 FBP | p<0.0001 | p<0.0001 | p<0.0001 | | p<0.0001 | | p<0.0001 | p<0.0001 |
| 240 FBP | 120 FBP | p<0.05 | p<0.05 | p<0.05 | | p<0.05 | | p<0.01 | p<0.01 |
| 240 FBP | 60 FBP | p<0.0001 | p<0.0001 | p<0.0001 | | p<0.0001 | | p<0.0001 | p<0.0001 |
| 120 FBP | 60 FBP | p<0.05 | p<0.05 | p<0.05 | | p<0.05 | | p<0.05 | p<0.05 |
